# Antibodies to Cryptic Epitopes in Distant Homologues Underpin a Mechanism of Heterologous Immunity between *Plasmodium vivax* PvDBP and *Plasmodium falciparum* VAR2CSA

**DOI:** 10.1128/mBio.02343-19

**Published:** 2019-10-08

**Authors:** Catherine J. Mitran, Angie Mena, Sedami Gnidehou, Shanna Banman, Eliana Arango, Barbara A. S. Lima, Hazel Lugo, Aravindhan Ganesan, Ali Salanti, Anthony K. Mbonye, Francis Ntumngia, Khaled Barakat, John H. Adams, Flora S. Kano, Luzia H. Carvalho, Amanda E. Maestre, Michael F. Good, Stephanie K. Yanow

**Affiliations:** a School of Public Health, University of Alberta, Edmonton, Alberta, Canada; b Department of Biology, Campus Saint-Jean University of Alberta, Edmonton, Alberta, Canada; c Grupo Salud y Comunidad, Facultad de Medicina, Medellín, Antioquia, Colombia; d Fundação Oswaldo Cruz, Belo Horizonte, Minas Gerais, Brazil; e Faculty of Pharmacy and Pharmaceutical Sciences, University of Alberta, Edmonton, Alberta, Canada; f Department of Immunology and Microbiology, Centre for Medical Parasitology, University of Copenhagen, Copenhagen, Denmark; g School of Public Health, Makerere University College of Health Sciences, Kampala, Uganda; h College of Public Health, University of South Florida, Tampa, Florida, USA; i Institute for Glycomics, Griffith University, Southport, Queensland, Australia; j Department of Medical Microbiology and Immunology, University of Alberta, Edmonton, Alberta, Canada; NIAID/NIH

**Keywords:** *Plasmodium*, *vivax*, *falciparum*, VAR2CSA, PvDBP, heterologous immunity, cryptic epitopes, cross-species, epitopes, pregnancy, *Plasmodium falciparum*, *Plasmodium vivax*, malaria

## Abstract

Many pathogens evolve extensive genetic variation in virulence proteins as a strategy to evade host immunity. This poses a significant challenge for the host to develop broadly neutralizing antibodies. In Plasmodium falciparum, we show that a mechanism to circumvent this challenge is to elicit antibodies to cryptic epitopes that are not under immune pressure. We previously discovered that antibodies to the Plasmodium vivax invasion protein, PvDBP, cross-react with P. falciparum VAR2CSA, a distantly related virulence factor that mediates placental malaria. Here, we describe the molecular mechanism underlying this cross-species immunity. We identified an epitope in subdomain 1 (SD1) within the Duffy binding-like (DBL) domain of PvDBP that gives rise to cross-reactive antibodies to VAR2CSA and show that human antibodies affinity purified against a synthetic SD1 peptide block parasite adhesion to chondroitin sulfate A (CSA) *in vitro*. The epitope in SD1 is subdominant and highly conserved in PvDBP, and in turn, SD1 antibodies target cryptic epitopes in P. falciparum VAR2CSA. The epitopes in VAR2CSA recognized by vivax-derived SD1 antibodies (of human and mouse origin) are distinct from those recognized by VAR2CSA immune serum. We mapped two peptides in the DBL5ε domain of VAR2CSA that are recognized by SD1 antibodies. Both peptides map to regions outside the immunodominant sites, and antibodies to these peptides are not elicited following immunization with VAR2CSA or natural infection with P. falciparum in pregnancy, consistent with the cryptic nature of these target epitopes.

## Abstract (precis)

In this work, we describe a molecular mechanism of heterologous immunity between two distant species of *Plasmodium*. Our results suggest a mechanism that subverts the classic parasite strategy of presenting highly polymorphic epitopes in surface antigens to evade immunity to that parasite. This alternative immune pathway can be exploited to protect pregnant women from falciparum placental malaria by designing vaccines to cryptic epitopes that elicit broadly inhibitory antibodies against variant parasite strains.

## INTRODUCTION

Heterologous immunity can develop when prior exposure to one pathogen modulates the host immune response to pathogens of genetically diverse species. This form of immunity can be induced by vaccination or arise from natural infection and lead to protective or deleterious outcomes following infection with a different organism. One of the classic examples of heterologous protection is the success of the cowpox vaccine in eradicating human smallpox. More recently, vaccination with the meningococcal B vaccine correlated with reduced incidence of gonorrhea (1, 2), and natural exposure to the nontuberculous mycobacteria in the environment can induce heterologous immunity to Mycobacterium tuberculosis (3). This phenomenon can also be modeled in mice, where immunization or infection with one virus protects against heterologous viral challenge (4). For example, infection with influenza A virus can protect against challenge with respiratory syncytial virus (RSV) and vaccinia virus (5, 6) and immunization with adenovirus can protect against hepatitis C virus (7). Similarly, infection with Japanese encephalitis virus in mice can prime the immune response and promote rapid viral clearance following heterologous challenge with Zika virus (8).

Despite the evidence that cross-species immunity can be highly protective, it remains controversial whether this form of immunity plays a role in protection from malaria. Malaria is a parasitic disease caused by five species of *Plasmodium* with overlapping endemicity in many geographic areas. Population-based data from several regions where multiple species of *Plasmodium* coexist, such as South Asia, Southeast Asia and parts of Oceania, are consistent with heterologous immunity against Plasmodium falciparum following prior infection with Plasmodium vivax (9–13). However, the mechanism of heterologous immunity to malaria is not defined. In humans, T cells induced by vaccination with P. falciparum respond *in vitro* to Plasmodium knowlesi-infected red blood cells, suggesting a role for the cellular arm of the immune system (14). In other studies, there is clearly heterologous cross-reactivity between antibodies as sera from patients infected with P. vivax cross-reacted with antigens from P. falciparum (15–19). These antibodies may cross-react with orthologous proteins involved in the same biological pathways in each species, such as PfMSP5/PvMSP5 (20), PfCLAG9/PvCLAG9 (21), PfAMA-1/PvAMA-1 (22), PfCSP/PvCSP and PfMSP-1/PvMSP-1 (23), and between Pfs48/45 and Pvs48/45 (24). However, functional activity of these cross-reactive antibodies was not reported.

We recently identified cross-species immune recognition between two homologous parasite proteins that are implicated in distinct biological pathways—P. vivax DBP (PvDBP) and P. falciparum VAR2CSA (25). PvDBP is a protein expressed on the surface of merozoites that mediates invasion into red blood cells by binding to the Duffy antigen receptor for chemokines (DARC) (26). VAR2CSA, on the other hand, is expressed by mature P. falciparum trophozoites and mediates sequestration of infected red blood cells (iRBCs) in the placenta (27–30). Sequestration is a common immune evasion mechanism thought to prevent destruction of iRBCs in the spleen and involves the interaction between certain antigenic variants of the P. falciparum erythrocyte membrane protein 1 (PfEMP1) family expressed on the surface of iRBCs with specific host receptors in different tissues (31). In pregnancy, parasites express the PfEMP1 variant VAR2CSA, which binds to chondroitin sulfate A (CSA) in the placenta. Placental sequestration is an important feature of malaria in pregnancy that can lead to poor outcomes for mother and baby, including stillbirth, preterm birth, low birthweight, and maternal anemia and death (32–34).

Most studies that evaluated the acquisition of VAR2CSA antibodies have focused on women in sub-Saharan Africa, where P. falciparum is the dominant species (35). These studies showed parity-dependent anti-VAR2CSA antibody acquisition, which occurred following multiple malaria infections in pregnancy (29). We discovered an alternate route of anti-VAR2CSA antibody acquisition outside pregnancy in areas where P. vivax and P. falciparum cocirculate (25). We showed that cross-reactivity is mediated by Duffy binding-like (DBL) domains, which are structurally conserved domains present in many *Plasmodium* proteins, including VAR2CSA, which has 6 DBL domains (30), and PvDBP, which has one (36). Antibodies against the DBL domain in region II of PvDBP (DBPII) from nonpregnant populations exposed to P. vivax recognized VAR2CSA by enzyme-linked immunosorbent assay (ELISA). Moreover, a mouse monoclonal antibody (MAb) against DBPII recognized VAR2CSA and blocked parasite adhesion to CSA *in vitro*.

Here, we probed the underlying mechanism of heterologous immunity to VAR2CSA. We identified a subdominant epitope in DBPII that mediates cross-reactivity to VAR2CSA and show that human antibodies purified against this epitope block iRBC adherence to CSA. Furthermore, both the human epitope-specific antibodies and the mouse MAb recognize overlapping, cryptic epitopes in VAR2CSA.

## RESULTS

### SD1ss contains the epitope in DBPII that is recognized by 3D10 and mediates cross-reactivity to VAR2CSA.

**FIG 1:**
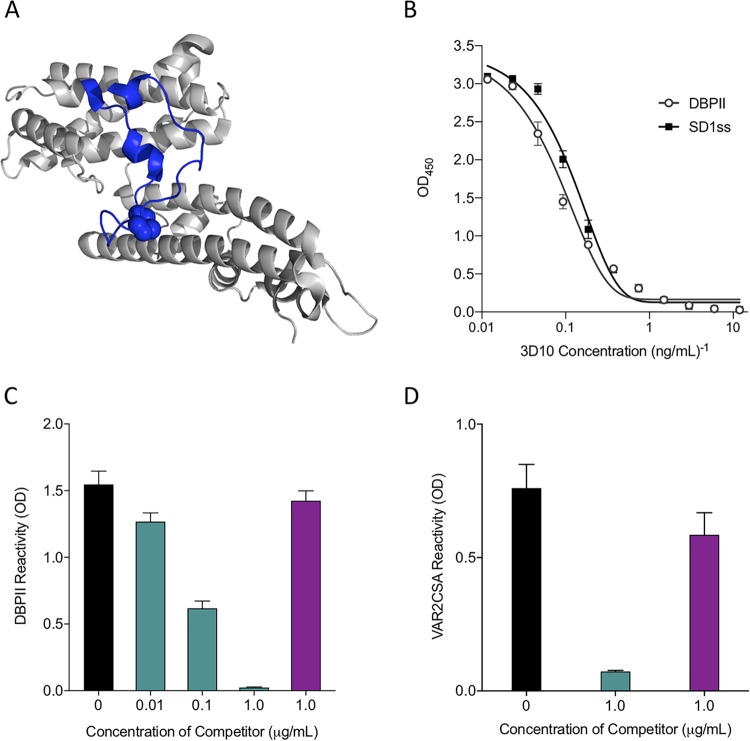
SD1ss contains an epitope in DBPII that is recognized by 3D10 and mediates cross-reactivity to VAR2CSA. (A) DBPII crystal structure (PDB ID 4NUU) showing the SD1 region in blue. The disulfide bond in SD1ss is represented by space-filling spheres. (B) Titration of 3D10 against recombinant DBPII and synthetic SD1ss. (C and D) 3D10 was incubated alone (black bar) or with SD1ss (teal bars) or the shorter peptide C_29_-K_40_ (purple bars) before being added to wells coated with DBPII (C) or VAR2CSA (D). Data are mean ± standard deviation (SD).

We showed previously that the 3D10 MAb against DBPII cross-reacted with VAR2CSA and blocked parasite adhesion to CSA *in vitro* (25). The epitope recognized by 3D10 is predicted to localize to subdomain 1 (SD1) of DBPII based on mutational analysis of this domain and peptide library screening with the MAb (37, 38). We designed a synthetic peptide, SD1ss, which spans the 39-amino-acid SD1 sequence and mutated the two outer cysteine residues to serines to ensure formation of a single disulfide bond (Fig. 1A). We confirmed 3D10 recognition of SD1ss by ELISA and found that 3D10 had the same endpoint titer against DBPII and SD1ss (0.17 ng/ml) (Fig. 1B).

We then tested the ability of the SD1ss peptide to block 3D10 recognition of DBPII using a competition ELISA. In this assay, 3D10 was incubated with increasing concentrations of SD1ss and then added to wells coated with DBPII (Fig. 1C). As a negative control, 3D10 was incubated with C_29_-K_40_ (at 1.0 μg/ml), a short peptide within SD1 that is not recognized by 3D10 (see Fig. S1 in the supplemental material). SD1ss blocked recognition of DBPII by 3D10 at a concentration of 1.0 μg/ml (Fig. 1C), confirming that this peptide contains the epitope that mediates recognition of DBPII. Next, we performed a competition ELISA with full-length recombinant VAR2CSA as the capture antigen. When 3D10 was incubated with SD1ss (at 1.0 μg/ml), but not C_29_-K_40_, recognition of VAR2CSA was blocked (Fig. 1D), showing that SD1ss contains an epitope that mediates cross-reactivity to VAR2CSA.

10.1128/mBio.02343-19.1 FIG S1 Peptide C_29_-C_40_ is not recognized by 3D10. The 3D10 MAb did not recognize the C_29_-C_40_ peptide within SD1. Download FIG S1, TIF file, 2.8 MB. Copyright © 2019 Mitran et al. 2019 Mitran et al. This content is distributed under the terms of the Creative Commons Attribution 4.0 International license.

### SD1ss is a subdominant epitope in PvDBP.

**FIG 2:**
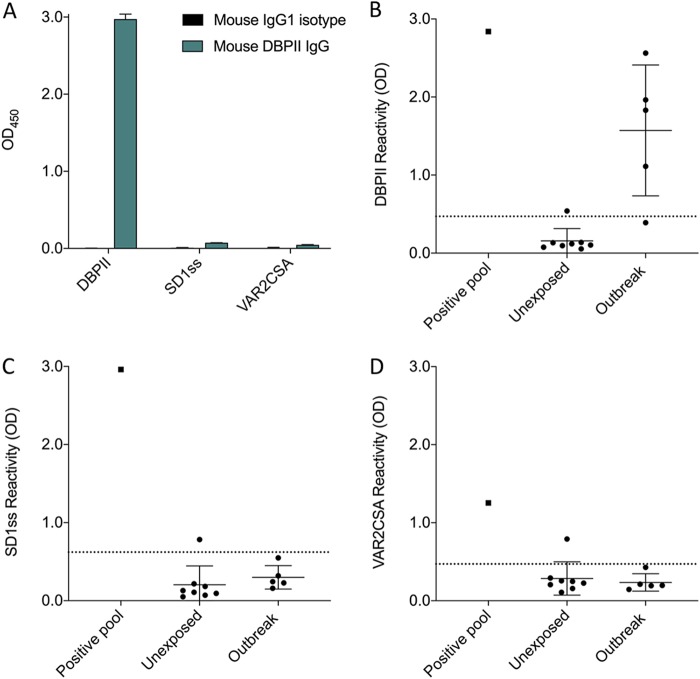
The SD1 domain of DBPII is subdominant. (A) Total IgG pooled from 15 mice immunized with the Sal 1 allele of DBPII was tested against DBPII, SD1ss, and VAR2CSA by ELISA. IgG was tested at a concentration of 0.1 μg/ml against DBPII and 5.0 μg/ml against SD1ss and VAR2CSA. (B to D) Sera from individuals infected during an outbreak of P. vivax in a malaria-free region of Brazil were tested by ELISA against DBPII (B), SD1ss (C), and VAR2CSA (D). The cutoff was defined as 2 standard deviations above the mean OD of unexposed Brazilians tested against the same antigens. A pool of sera from individuals living in an area of malaria endemicity in Brazil was included as a positive control. Data are mean ± SD.

To test whether SD1ss is a dominant epitope in PvDBP, we generated and tested polyclonal sera to DBPII in BALB/c mice using the same strain of mice and the same allele of DBPII (Sal 1) that gave rise to the 3D10 MAb. Total IgG from mice immunized with Sal 1 DBPII recombinant protein did not recognize SD1ss by ELISA, despite high antibody titers to DBPII (Fig. 2A). These antibodies also failed to recognize full-length VAR2CSA. These data demonstrate that SD1 is subdominant in this strain of mouse and are consistent with the observation that of 7 MAbs generated to DBPII, only 3D10 recognizes the SD1 region (37, 38).

To investigate this in a human population, we tested sera from individuals infected during a P. vivax outbreak in Brazil that occurred in an otherwise malaria-free region (39). Sera from individuals who experienced P. vivax relapses developed a range of DBPII antibodies (Fig. 2B) but failed to recognize SD1ss (Fig. 2C). These human sera did not recognize VAR2CSA (Fig. 2D), consistent with the mouse data that polyclonal antibodies against DBPII that lack specificity for SD1ss do not cross-react with VAR2CSA. It is possible that these sera failed to recognize SD1ss due to polymorphisms between the peptide sequence and the SD1 sequence in the outbreak clone. However, when we sequenced the SD1 region from P. vivax genomic DNA (gDNA) isolated from a patient during the outbreak, the sequence was 100% identical to SD1 in Sal 1, the strain used to design the SD1ss peptide. Furthermore, a BLASTp search of the SD1 amino acid sequence showed that it was 100% identical to the top 100 P. vivax SD1 sequences from global isolates, suggesting that this region of DBPII is highly conserved and not under immune selection.

**FIG 3:**
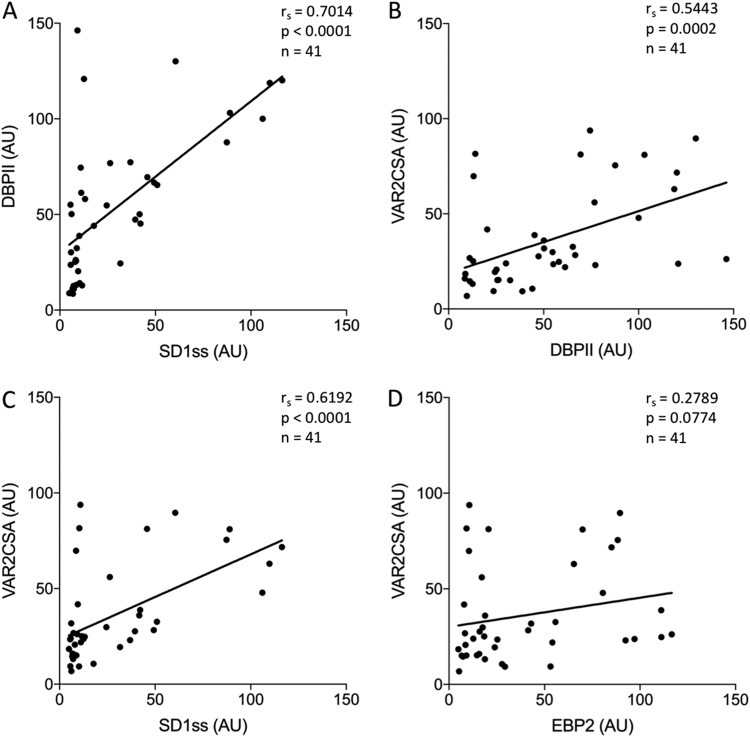
SD1ss antibodies correlate with VAR2CSA antibodies in sera from Brazilian men and children with exposure to P. vivax only. Individual sera from men and children from Rio Pardo, Brazil, were selected based on past exposure to P. vivax only. Sera were tested by ELISA for reactivity to DBPII, SD1ss, VAR2CSA, and EBP2. Antibody levels (ODs) were converted to arbitrary units (AU) based on the positive control included on every plate. Serum reactivity was correlated using Spearman rank correlation. *r_s_*, Spearman rank coefficient.

We showed previously that P. vivax-exposed Brazilian men and children residing in Rio Pardo, an area of malaria endemicity in the Amazon, had antibodies against VAR2CSA (25). Based on this finding, and the data above, we postulated that the SD1 epitope in PvDBP is poorly immunogenic. To address this, we tested sera from Brazilian men and children with lifelong exposure to P. vivax for antibodies to SD1ss and DBPII. We observed that 78% had antibodies to DBPII whereas only 39% had antibodies to SD1ss. The antibody levels were correlated (*r_s_* = 0.7014, *P* < 0.0001) (Fig. 3A), but half of those who had DBPII antibodies did not have SD1ss antibodies. These findings are consistent with the mouse and outbreak data showing that exposure to DBPII does not always elicit an antibody response against SD1ss and confirm that this epitope in PvDBP is subdominant in human populations.

We then correlated the seroreactivity to DBPII and SD1ss with VAR2CSA reactivity and found that VAR2CSA antibody levels were correlated with both DBPII (*r_s_* = 0.5443, *P* = 0.0002) and SD1ss (*r_s_* = 0.6192, *P* < 0.0001) (Fig. 3B and C). To assess the specificity of these interactions, the sera were tested against another P. vivax merozoite protein, EBP2. This protein is a homologue of DBPII, but the SD1-like region is significantly different from DBPII, and the 3D10 MAb does not recognize this protein (40). Antibody levels against EBP2 did not correlate with VAR2CSA reactivity in this population (*r_s_* = 0.2789, *P* = 0.0774) (Fig. 3D).

**FIG 4:**
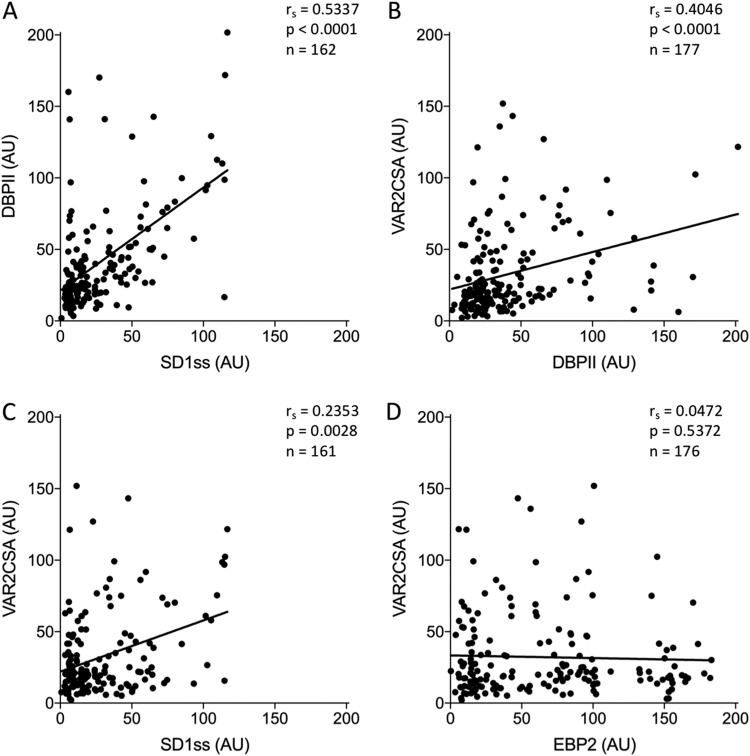
SD1ss antibodies correlate with VAR2CSA antibodies in sera from Colombian men and children. Sera from men and children living in an area of malaria endemicity in Colombia were tested for reactivity to DBPII, SD1ss, VAR2CSA, and EBP2 by ELISA. Antibody levels (ODs) were converted to arbitrary units (AU) based on the positive control included on every plate. Serum reactivity was correlated using Spearman rank correlation. *r_s_*, Spearman rank coefficient.

We observed similar correlations of antibody levels in sera from men and children living in an area of Colombia where both P. vivax and P. falciparum are endemic (Fig. 4). These subjects were not selected based on past or current malaria infection and represent a more heterogeneous population in terms of malaria exposure. DBPII recognition was significantly correlated with SD1ss antibody levels (*r_s_* = 0.5337, *P* < 0.0001), and VAR2CSA antibodies correlated with both DBPII (*r_s_* = 0.4046, *P* < 0.0001) and SD1ss (*r_s_* = 0.2353, *P* = 0.0028) antibody levels (Fig. 4A to C). Again, there was no correlation between EPB2 and VAR2CSA antibody levels (*r_s_* = 0.0472, *P* = 0.5372) (Fig. 4D).

### SD1ss affinity-purified human antibodies block parasite adhesion to CSA *in vitro*.

**FIG 5:**
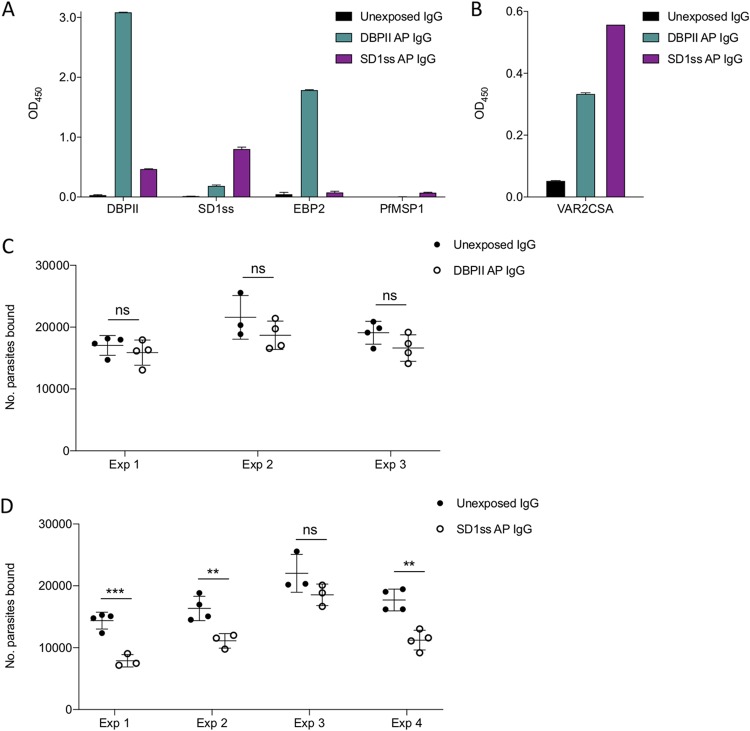
SD1ss affinity-purified antibodies recognize VAR2CSA and block iRBC adhesion to CSA. (A and B) Pooled sera from Colombian men and children were affinity purified on DBPII or SD1ss. Total IgG purified from unexposed Colombians was used as a negative control. Antibodies were tested against various antigens by ELISA. (C and D) Affinity-purified DBPII (100 μg/ml) (C) and SD1ss (90 μg/ml) (D) antibodies were tested in the inhibition-of-binding assay using P. falciparum CS2 parasites expressing VAR2CSA. Data are from independent experiments. Data are mean ± SD, and significance was determined using Student’s *t* test comparing unexposed Colombian IgG and affinity-purified IgG for each experiment. **, *P* < 0.01; ***, *P* < 0.001; ns, not significant.

Our data thus far suggest that antibodies to the epitope in PvDBP SD1 cross-react with VAR2CSA. To test this directly, we affinity purified antibodies that recognize SD1ss from a pool of sera from nonpregnant populations in Colombia (men and children) exposed to P. vivax and P. falciparum. Similarly, we affinity purified antibodies that recognize the entire DBPII recombinant protein. As expected from our immunogenicity data above, the DBPII affinity-purified antibodies recognized SD1ss very weakly (Fig. 5A). They did recognize EBP2, presumably through shared epitopes in the DBL domain. In contrast, the SD1ss affinity-purified antibodies recognized DBPII but not EBP2, consistent with poor sequence conservation of the SD1-like region in EBP2. Neither of the purified antibodies recognized PfMSP1, an unrelated P. falciparum merozoite antigen. Both affinity-purified antibodies cross-reacted with VAR2CSA, and the reactivity of the SD1ss affinity-purified antibodies was higher than the DBPII affinity-purified antibodies (Fig. 5B).

A critical question is whether the affinity-purified antibodies could protect against placental malaria. This can be measured *in vitro* using an inhibition-of-binding assay (IBA) to test if antibodies block parasite adhesion to CSA. Mature VAR2CSA-expressing P. falciparum CS2 trophozoites were incubated with the affinity-purified IgG and then added to immobilized CSA. The number of parasites bound per spot of CSA was quantified and compared to binding in the presence of IgG from a pool of unexposed Colombians as the negative control. We found that the DBPII affinity-purified IgG reduced parasite binding to CSA, but the effect was not significant (Fig. 5C). However, SD1ss affinity-purified IgG blocked parasite adhesion to CSA, and the effect was significant in three of four experiments (Fig. 5D). The inhibition in these three experiments ranged from 32 to 45%.

### Antibodies to SD1 in PvDBP target cryptic epitopes in VAR2CSA.

**FIG 6:**
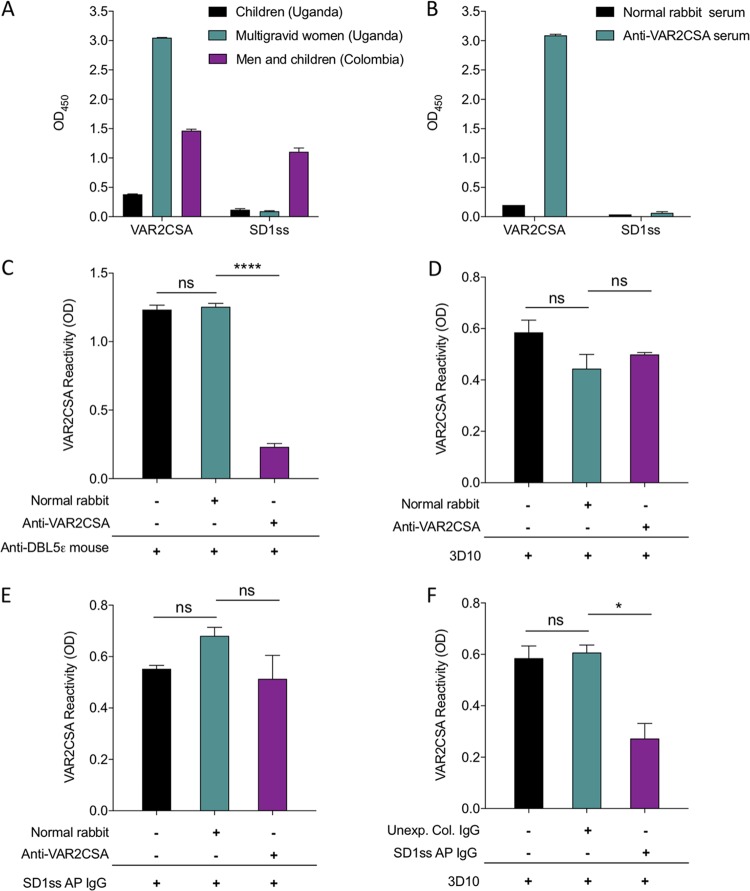
Epitopes in VAR2CSA recognized by vivax-derived antibodies are cryptic. (A) Pooled sera from Ugandan children and multigravid women were tested by ELISA against VAR2CSA and SD1ss. Sera from Colombian men and children were used as a positive control. (B) Anti-VAR2CSA rabbit serum were tested by ELISA against VAR2CSA and SD1ss. (C to F) Competition ELISAs with VAR2CSA as the capture antigen and either anti-VAR2CSA rabbit serum (C to E) or human SD1ss affinity-purified antibodies (F) as the competing antibody. 3D10 (D and F) or human SD1ss affinity-purified antibodies (E) were added as the source of detection antibodies. Data are mean ± SD, and significance was determined using a one-way ANOVA with multiple-comparison test. *, *P* < 0.05; ****, *P* < 0.0001; ns, not significant; AP, affinity-purified.

To better understand this mechanism of cross-species immunity, we investigated whether antibodies that developed following exposure to P. falciparum VAR2CSA would reciprocally recognize the epitope in PvDBP SD1. We first tested plasma collected from multigravid women from Uganda who were naturally exposed to VAR2CSA during pregnancy (Fig. 6A). Despite high levels of VAR2CSA-specific antibodies, there was no recognition of SD1ss. Pooled sera from Ugandan children were included as a negative control. Sera from Colombian men and children that recognized both VAR2CSA and SD1ss were included as a positive control. To investigate this further, we tested serum from a rabbit that was immunized with recombinant VAR2CSA. Similarly to the human sera, the rabbit serum did not recognize SD1ss (Fig. 6B).

These data imply that cross-reactive antibodies (elicited against SD1) and VAR2CSA-induced antibodies recognize distinct epitopes in VAR2CSA. To test this further, we performed an antibody-based competition ELISA using the 3D10 MAb and VAR2CSA antibodies induced through immunization (in rabbits). Initially, rabbit anti-VAR2CSA serum or normal rabbit serum (NRS) (as a control) was added to wells coated with VAR2CSA. Then, the detecting antibody (anti-DBL5ε mouse serum, 3D10 MAb, or human SD1ss affinity-purified antibodies) was added. The rabbit anti-VAR2CSA antibody strongly blocked recognition by the anti-DBL5ε serum, compared to the NRS (Fig. 6C), consistent with recognition of shared immunodominant epitopes by these sera. However, VAR2CSA rabbit antiserum could not block recognition of VAR2CSA by 3D10 (Fig. 6D), demonstrating that the epitope on VAR2CSA recognized by 3D10 was not recognized by the polyclonal rabbit antiserum. Similarly, the rabbit polyclonal antiserum could not block recognition of VAR2CSA by human SD1ss affinity-purified antibodies (Fig. 6E), demonstrating that, like 3D10, the epitope(s) on VAR2CSA recognized by human antibodies that arose as a result of P. vivax exposure is distinct from those that are immunogenic in VAR2CSA. Thus, the epitopes on VAR2CSA recognized by 3D10 and by humans following P. vivax exposure are classical cryptic epitopes. To ask whether the human and mouse cryptic epitopes are the same, we tested whether SD1ss affinity-purified human antibodies could block recognition of 3D10 (Fig. 6F). We demonstrated partial but significant blocking, suggesting that these epitopes overlap but may not be identical.

### Cryptic epitopes in the DBL5ε domain of VAR2CSA.

**FIG 7:**
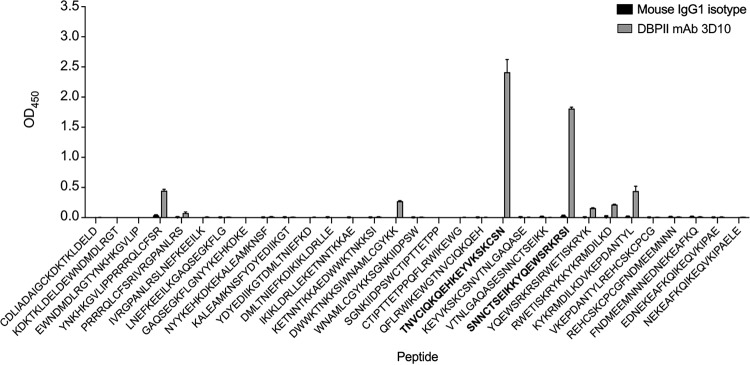
3D10 recognizes epitopes in DBL5ε. An array of overlapping peptides that span DBL5ε was tested with the DBPII MAb 3D10 compared with a mouse IgG1 isotype control. P20 and P23 are in bold.

**FIG 8:**
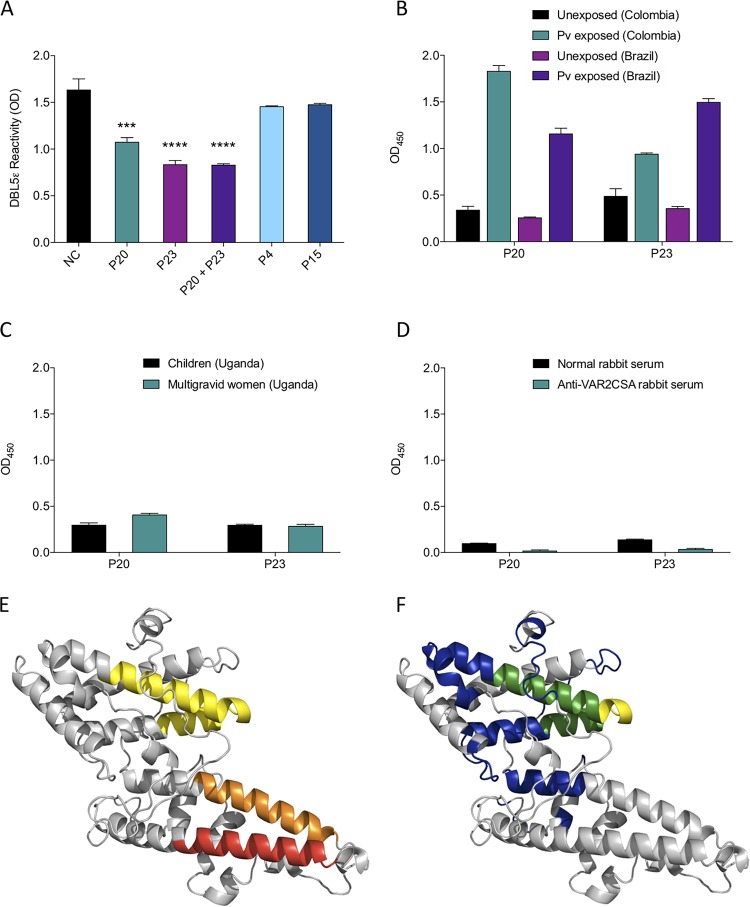
Recognition of specific peptides in the DBL5ε domain of VAR2CSA by vivax-derived antibodies. (A) Competition of 3D10 recognition of DBL5ε by individual peptides. 3D10 was incubated with peptides P20, P23, P20 and P23 in combination, P4, and P15 (all at 100 μg/ml) and added to a plate coated with DBL5ε protein. The OD of 3D10 preincubated with each peptide was compared to the OD for the antibody alone (no competitor). NC, no competitor. (B to D) Recognition of P20 and P23 by sera from Colombians and Brazilians exposed only to P. vivax (B), children and multigravid women from Uganda (C), and a rabbit immunized with full-length VAR2CSA (D) was tested by ELISA. (E) Homology model of DBL5ε depicting P20 (orange), P23 (red), and the putative glycosaminoglycan binding site (yellow) (42). (F) Homology model of DBL5ε depicting the surface-exposed regions recognized by sera from multigravid African women (blue), some of which overlap (green) the putative glycosaminoglycan binding site (yellow) (42). Data are mean ± SD, and significance was determined using a one-way ANOVA with multiple-comparison test (***, *P* < 0.001; ****, *P* < 0.0001).

VAR2CSA is a large multidomain protein that could contain many target epitopes for the cross-reactive antibodies derived from SD1. We focused on mapping the epitopes in one domain, DBL5ε, as this domain is among the more conserved DBL domains in VAR2CSA and we showed previously that this domain is strongly recognized by the 3D10 MAb (25). We generated an array of overlapping peptides that span the DBL5ε domain and screened this array with 3D10 (Fig. 7). Two peptides were strongly recognized (P20 and P23), while two others (P4 and P15) were weakly recognized. To validate these peptides further, we performed competition ELISAs and tested whether each peptide could compete out the recognition of DBL5ε by 3D10 (Fig. 8A). Only P20 and P23 significantly reduced the recognition by 3D10. While the effect with each peptide was partial, there was no synergistic effect of combining the two peptides (Fig. 8A).

We next determined whether the epitopes in P20 and P23 were cryptic in DBL5ε. Whereas the pools of sera from Colombian and Brazilian men and children exposed only to P. vivax strongly recognized these same peptides (Fig. 8B), they were not recognized by the sera from either Ugandan multigravid women (Fig. 8C) or the rabbit immunized with VAR2CSA (Fig. 8D).

To visualize the P20 and P23 epitopes within the protein structure of DBL5ε, we mapped the two peptide sequences onto a homology model of the DBL5ε (3D7) domain. Both peptides mapped to alpha helices in subdomain 3 (Fig. 8E). These sites are distinct from the immunodominant epitopes recognized by sera from Tanzanian multigravid women (Fig. 8F, blue) and a rabbit immunized with VAR2CSA (41) and share no overlap with the putative CSA binding sequence in this domain (Fig. 8E, yellow) (42).

P20 and P23 share only limited amino acid sequence homology with SD1ss; P20 contains two cysteine residues, while P23 contains one cysteine as well as the motif RKR, which is important for recognition of SD1 by 3D10 (37, 38). We therefore investigated the possibility that 3D10 recognized a conformational epitope in these peptides. To test this, we measured 3D10 reactivity to each peptide after treatment with dithiothreitol (DTT), which would abolish disulfide bonding either within or between peptide molecules. When P20 and P23 were treated with DTT, 3D10 recognition was lost (Fig. S2). Interestingly, 3D10 recognition of SD1ss was also reduced following treatment of the peptide with DTT, suggesting that the disulfide bond is important for recognition of the homologous epitopes.

10.1128/mBio.02343-19.2 FIG S2 3D10 recognizes conformational epitopes in P20, P23 and SD1ss. 3D10 recognition of P20 and P23 was lost when the peptides were treated with dithiothreitol (DTT). Recognition of SD1ss was reduced when the peptide was treated with DTT. Download FIG S2, TIF file, 2.8 MB. Copyright © 2019 Mitran et al. 2019 Mitran et al. This content is distributed under the terms of the Creative Commons Attribution 4.0 International license.

## DISCUSSION

We discovered a host defense mechanism in *Plasmodium* in which a subdominant epitope in the P. vivax antigen PvDBP elicits functional antibodies against cryptic epitopes in the distantly related P. falciparum homologue VAR2CSA. We mapped the epitope in PvDBP to SD1 and showed that human antibodies to this epitope recognized VAR2CSA and blocked parasite adhesion to CSA. Our data suggest that SD1 is subdominant in PvDBP by virtue of its poor immunogenicity in mice vaccinated with the Sal 1 allele of PvDBP and our findings that about half of individuals exposed to PvDBP do not develop SD1 antibodies. However, the levels of SD1 antibodies correlated with the levels of VAR2CSA reactivity. This is consistent with findings from another study where volunteers were deliberately infected with the P. vivax Sal 1 strain and did not have cross-reactive antibodies to VAR2CSA (43). We expect that the Sal 1 strain of PvDBP does not readily elicit SD1 antibodies in humans, as we observed in mice. While the mechanism that gives rise to SD1-specific antibodies during natural P. vivax infection is not known, certain alleles of PvDBP may adopt protein structures that preferentially expose SD1, or perhaps SD1 antibodies arise through affinity maturation following exposure to multiple, different PvDBP alleles. Alternatively, host genetic variability in HLA class II genes may also play a role in directing the antibody responses, as we reported previously for DBPII (44).

Antibodies to SD1 of PvDBP appear to have no protective value against P. vivax infection. The epitope is weakly immunogenic, and antibodies that do arise, such as 3D10 in mice, do not inhibit binding to DARC or block P. vivax invasion of reticulocytes (45). Moreover, the amino acid sequence of SD1 is highly conserved, which is consistent with our interpretation that this subdomain is not under immune pressure. Likewise, our data strongly suggest that antibodies to SD1 target cryptic epitopes in VAR2CSA. Immune serum from a rabbit immunized with VAR2CSA failed to compete with the human SD1ss affinity-purified antibodies or the mouse 3D10 MAb for recognition sites on VAR2CSA. Yet, the binding of SD1 antibodies to these cryptic epitopes can block parasite adhesion to CSA. We do not know whether the epitopes recognized by the vivax-derived antibodies are within the CSA binding sites of VAR2CSA or if the antibodies block adhesion by steric hindrance. However, the two peptides that we identified in the DBL5ε array map to sites distal to the predicted CSA binding sequence in this domain (42). They are also distinct from the immunodominant epitopes that were recognized by African multigravid women (41). Further work is needed to identify the target epitopes in other DBL domains of VAR2CSA and how they map to the three-dimensional structure of the full protein.

To our knowledge, this is the first demonstration of adhesion-blocking antibodies that target cryptic epitopes in VAR2CSA. Antibodies can access cryptic epitopes through various mechanisms. Epitopes concealed within the head domain of the influenza A hemagglutinin protein are transiently exposed to antibodies during dynamic changes in the conformation of the trimer complex, through a process described as “breathing” (46–48). Similarly, several studies on dengue virus and West Nile virus revealed time- and temperature-dependent exposure of cryptic epitopes, reflecting the important role of structural dynamics in epitope accessibility (49–52). VAR2CSA forms a large globular complex that may undergo similar protein dynamics, exposing cryptic epitopes within intermediate protein conformations that arise during transport to the cell surface, posttranslational modification/protein maturation, interaction with other knob proteins, or upon binding to CSA. The idea that these epitopes are exposed in only a subset of protein structures is consistent with our previous finding that the 3D10 MAb recognized only a small percentage of iRBCs that expressed native VAR2CSA on the cell surface (25).

The discovery of cryptic epitopes in VAR2CSA has direct applications for vaccine development against placental malaria. A significant obstacle to current vaccine strategies is the highly polymorphic nature of *var2csa* alleles (53), which impedes development of broadly neutralizing antibodies against diverse parasite strains. In a recent study of 247 P. falciparum isolates (54), 171 polymorphic loci were identified in the ID1-DBL2Xb subregion that is included in two vaccines against pregnancy-associated malaria (55, 56). A different study identified 4 major domain clades in this region (57). The effects of these polymorphisms are highlighted by several reports that functional antibodies to VAR2CSA in different populations of African women are highly strain specific, reflecting seroreactivity to immunodominant epitopes (58–60). Our finding that SD1 from P. vivax elicits protective antibodies against cryptic epitopes in VAR2CSA provides an alternative, yet complementary, vaccine strategy that could circumvent this immune pressure.

Epitope-specific vaccines that direct the immune response to cryptic or subdominant epitopes are emerging as a viable strategy against many pathogens for which traditional vaccine approaches were unsuccessful (61, 62). These vaccine candidates include conserved epitopes in otherwise highly mutable viruses, such as HIV (63, 64), Ebola virus (65, 66), and influenza virus (46–48, 67, 68), as well as pathogenic bacteria, such as Streptococcus pyogenes (69), anthrax and Staphylococcus aureus (61). A critical advantage is that many of these epitopes can induce broadly neutralizing, strain-transcending immunity by eliciting antibodies that cross-react with related but antigenically distinct pathogens (66, 69, 70).

In summary, we show that antibodies to a highly conserved, subdominant epitope in P. vivax inhibit an unrelated virulence pathway in P. falciparum *in vitro*. It will be important in future clinical studies to determine whether these antibodies contribute to improved birth outcomes from P. falciparum placental malaria and to evaluate whether SD1 can elicit polyclonal neutralizing antibodies through immunization. While our data make SD1 an attractive epitope for future vaccine design, they also provide insight into an unusual mechanism of heterologous immunity to *Plasmodium* based on shared epitopes across the DBL protein family.

## MATERIALS AND METHODS

### Human subjects.

Approval for this study was granted by the Health Research Ethics Board of the University of Alberta in Canada (approval Pro00041720); the Comité de Ética of the Instituto de Investigaciones Médicas of the Universidad de Antioqua in Colombia (approvals 009-2013, 002-2015, and 009-2016); the Ethics Committee of the Fundação Oswaldo Cruz, the Brazilian Health Ministry, and the Ethical Committee of Research on Human Beings from the CPqRR/Fundação Oswaldo Cruz (reports 07/2009 and 26/2013; CAEE:50522115.7.0000.5091/05/2016); and the Higher Degrees, Research, and Ethics Committee in Uganda (HDREC approval 386). Participation in all studies was voluntary, and each participant provided written consent.

Samples from individuals in Colombia, Brazil, and Uganda were included in this study. In Souza, Brazil, samples were collected from individuals who were infected during a P. vivax outbreak in an otherwise malaria-free region (39). Samples from five individuals were tested in this study. Malaria-exposed individuals were recruited from the agricultural settlement of Rio Pardo, in the Brazilian Amazon, to participate in a population-based open cohort study initiated in November 2008 (71). In this study, we included samples from individuals who were positive for exposure to P. vivax (based on PvMSP1 reactivity) but negative for P. falciparum exposure (based on PfMSP1 reactivity). Samples were also collected from unexposed Brazilians living in Belo Horizonte and used as negative controls. In Colombia, both symptomatic and asymptomatic men and children (including girls under 12 years of age) were recruited between 2013 and 2016 in the municipality of Puerto Libertador in the Department of Córdoba. Serum samples were collected in the community as part of a cross-sectional survey (asymptomatic cohort) and from individuals presenting to the clinic with suspected malaria (25). Serum samples from 50 unexposed individuals living in Medellín, Colombia, were also collected and used as negative controls. In Bugiri, Uganda, plasma samples were collected from individuals over 1 year of age who presented to the clinic with suspected malaria, as part of another study (72). The samples used in this study were collected from male children and multigravid women, who were pregnant at the time of collection.

### Mice.

Mice used for the Sal 1 immunizations were purchased from Harlan Animal Research Laboratories and housed in the University of South Florida Animal Facility. Female BALB/c mice (6 to 8 weeks old) were used for immunizations. All procedures were approved by the Institutional Animal Care and Use Committee. Mice used for the DBL5ε immunizations were purchased from Charles River Laboratories and housed in the University of Alberta Animal Facility in a virus antibody-free room. Female BALB/c mice (6 to 8 weeks old) were used for immunizations. All procedures were approved by the University of Alberta Animal Care and Use Committee, and mice were handled in accordance with the Canadian Council on Animal Care guidelines.

### Synthetic peptide design.

The SD1ss peptide was synthesized (Synpeptides Co.) with the following sequence: ASNTVMKNSNYKRKRRERDWDCNTKKDVCIPDRRYQLSMK. In this peptide, two of the cysteines (C_9_ and C_38_) were mutated to serine to ensure that only one disulfide bond could form. Thirty-one overlapping 20-mer peptides were designed to cover the entire DBL5ε domain of VAR2CSA (Mimotopes).

### ELISAs.

For indirect ELISAs, 96-well plates (catalogue no. 439454; Thermo Fisher Scientific) were coated with antigen in 1× phosphate-buffered saline (PBS) overnight at 4°C (antigen concentrations are listed in Table S1 in the supplemental material). For ELISAs using peptides treated with DTT, the peptides were incubated with DTT (10 mM) at 56°C for 10 min and then added to the plate. Wells were blocked with 4% bovine serum albumin (BSA) (catalogue no. A7906; Sigma-Aldrich) for 1 h at 37°C followed by incubation with the primary antibody for 1 h at room temperature (RT) (antibody dilutions are listed in Table S1). After four washes with 1× PBST (0.01% Tween 20), a horseradish peroxidase (HRP)-conjugated secondary antibody was added and the plate was incubated for 1 h at RT (secondary antibody dilutions are listed in Table S1) (goat anti-rabbit HRP, catalogue no. 65-6120 [Invitrogen]; goat anti-mouse HRP, catalogue no. 170-6516 [Bio-Rad]; goat anti-human HRP, catalogue no. ab98624 [Abcam]). The plate was again washed four times with 1× PBST before the developing reagent (3,3′,5,5′-Tetramethylbenzidine [TMB], catalogue no. T0440; Sigma-Aldrich) was added to each well. The reaction was stopped after 30 min at RT by addition of an equal amount of sulfuric acid (0.5 N), and the optical density (OD) of each well was measured at 450 nm. All samples were run in duplicate, and the average OD for the antigen plus secondary antibody alone was subtracted from the OD of all samples.

10.1128/mBio.02343-19.3 TABLE S1 ELISA reagent details. Download Table S1, PDF file, 0.1 MB. Copyright © 2019 Mitran et al. 2019 Mitran et al. This content is distributed under the terms of the Creative Commons Attribution 4.0 International license.

Peptide competition ELISAs were performed as described for indirect ELISAs, except that the primary antibody was first incubated with a test peptide (SD1ss, 0.01, 0.1, and 1.0 μg/ml; all other peptides, 100 μg/ml) for 30 min at RT before being added to the plate.

Antibody competition ELISAs were also performed as described for the indirect ELISA, except that a competing antibody was added to the plate after blocking. The plate was then washed four times with 1× PBST, and the detecting antibody was added. Following another set of washes, an HRP-conjugated secondary antibody directed against the detecting antibody was added.

### Immunization scheme.

For the DBPII (Sal 1 allele) immunizations, 15 female BALB/c mice (6 to 8 weeks old) were immunized as previously described (73). Mice were given three doses of recombinant DBPII (Sal 1 allele) (25 μg/mouse) emulsified in TiterMax Gold (catalogue no. T2684; Sigma) subcutaneously (s.c.) at days 0, 21, and 42. The final serum samples were collected 3 weeks after the last immunization.

For the DBL5ε immunizations, a female BALB/c (6 to 8 weeks old) mouse was immunized s.c. with recombinant DBL5ε in 2% Alhydrogel (CAS no. 21645-51-2; Brenntag Biosector) at day 0 (30 μg/mouse), day 21 (10 μg/mouse), and day 31 (10 μg/mouse), and serum was collected on day 45.

### Sequencing of Brazilian isolate.

Genomic DNA was extracted from 300 μl of whole blood using a genomic DNA purification kit (Puregene; Gentra Systems), according to the manufacturer’s protocol. The DBPII region was amplified using the following primers: 5′-CCGTTATGAAGAACTGCAACTACA-3′ and 5′-GAATGTGGCGGTGAATATCGAA-3′. The PCR product was isolated using the QIAquick PCR purification kit (catalogue no. 28104; Qiagen) and submitted for Sanger sequencing using the same primers used for PCR amplification.

### Affinity purification.

DBPII affinity purifications were performed using *N*-hydroxysuccinimide (NHS)-activated Sepharose beads (catalogue no. 17-0906-01; GE Healthcare) according to the manufacturer’s guidelines. Beads (1.0 ml) were added to a filter column and washed with 13 ml ice-cold HCl (1 mM). Recombinant DBPII (1.7 mg) dissolved in coupling buffer (200 mM NaHCO_3_, 500 mM NaCl, pH 8.3) was added to the column and incubated overnight at 4°C. Flowthrough was collected following centrifugation for 2 min at 500 × *g* for analysis of coupling efficiency. Any remaining active sites were deactivated by incubating the column for 2 h at RT with 2 ml of deactivation buffer (500 mM ethanolamine, 500 mM NaCl, pH 8.3). The column was then washed with 3 ml buffer 1 (100 mM Tris-HCl, 500 mM NaCl, pH 8.3), followed by 3 ml of buffer 2 (100 mM sodium acetate, 500 mM NaCl, pH 4.0). These washes were repeated three times before 5 ml of binding buffer (1× PBS) was flowed through the column. The column was prepared by washing with 3 ml elution buffer (100 mM glycine, pH 2.0), followed by 15 ml 1× PBS. Sera from men and children in Colombia exposed to P. vivax and P. falciparum were pooled (5 ml), diluted 1:1 in 1× binding buffer, clarified using an 0.45-μm filter, and then loaded onto the column and incubated for 30 min at RT on a rocker. The column was washed with 9 ml of binding buffer until no protein was detected in the flowthrough (measured using a NanoDrop spectrophotometer). Bound antibodies were then eluted using 5 ml of elution buffer into tubes containing an equal volume of neutralization buffer (1 M Tris-HCl, pH 9.0).

SD1ss affinity purifications were performed using NHS-activated HiTrap columns (catalogue no. 17-0716-01; GE Healthcare Life Sciences) according to the manufacturer’s guidelines. Columns were acidified with HCl (1 mM) and coated with 1.0 mg of SD1ss in coupling buffer (200 mM NaHCO_3_, 500 mM NaCl, pH 8.3). Unbound peptide or protein was then washed from the column using 3 column volumes of coupling buffer. Any remaining active sites were deactivated by flowing 2 ml of buffer A (500 mM ethanolamine, 500 nM NaCl, pH 8.3) through the column, followed by 2 ml of buffer B (100 mM acetate, 500 mM NaCl, pH 4.0) and then another 2 ml of buffer A. This was repeated six times, with a 30-min incubation in buffer A at RT after the third set of buffers was added. The column was then washed with 5 ml of 1× PBS, followed by 3 ml of elution buffer (0.1 M glycine-HCl, pH 2.0) and then another 10 ml of 1× PBS. Sera from men and children in Colombia exposed to P. vivax and P. falciparum were pooled (5 ml), diluted 1:1 in 1× PBS, clarified using an 0.45-μm filter, and loaded onto the column. The sample was continuously run over the column at a flow rate of approximately 0.5 ml/min for 1 h. The column was then washed with 1× PBS until no protein was detected in the flowthrough (measured using a NanoDrop spectrophotometer). Bound antibodies were then eluted using 5 ml of elution buffer into tubes containing an equal volume of neutralization buffer (1 M Tris-HCl, pH 9.0).

Elution fractions from each affinity purification that contained protein were pooled, and the buffer was exchanged with 1× PBS using an Amicon Ultra-4 centrifugal filter (catalogue no. UFC801024; Merck Millipore). Total IgG was then purified, and the concentration was measured using a NanoDrop spectrophotometer.

### Purification of total IgG.

Total IgG was purified from affinity-purified antibodies, pooled plasma, or serum samples using a HiTrap Protein G HP column (catalogue no. 17-0404-03; GE Healthcare Life Sciences) according to the manufacturer’s instructions. Briefly, the column was washed with 20 ml of 1× PBS before 1 ml of serum, plasma, or affinity-purified antibodies was loaded onto the column. The column was then incubated for 1 h at RT. The column was then washed with 1× PBS until there was no protein detected in the flowthrough (measured using a NanoDrop spectrophotometer). Bound IgG was eluted using 3 ml of elution buffer (0.1 M glycine-HCl, pH 9.0) into tubes containing an equal volume of neutralization buffer (1 M Tris-HCl, pH 9.0). Elution fractions containing protein were pooled, the buffer was exchanged with 1× PBS using an Amicon Ultra-4 centrifugal filter, and the concentration was measured using a NanoDrop spectrophotometer.

### P. falciparum culture.

P. falciparum CS2 parasites were maintained in culture at 3% hematocrit in washed erythrocytes collected from O^+^ blood donors as described previously (74). Parasites were regularly selected for adhesion to CSA (catalogue no. C9819; Sigma-Aldrich) to enrich for parasites expressing VAR2CSA. Mature parasites were magnetically purified using the VarioMACS separator according to the manufacturer’s instructions (LD columns, catalogue no. 130-042-901; Miltenyi Biotec).

### Inhibition of binding assay (IBA).

Ten spots were drawn in a semicircle around the outer edge of the bottom of a Petri dish (catalogue no. 351029; Corning). Each spot was coated with 20 μl CSA in 1× PBS (50 μg/ml) overnight at 4°C in a humidified chamber. The spots were then blocked with 3% BSA in RPMI (catalogue no. 31800-022; Gibco Life Technologies) for 1 h at 37°C in a humidified chamber. Mature trophozoite-stage P. falciparum CS2 parasites were magnetically enriched using the VarioMACS separator and diluted to 1.0 × 10^7^ cells/ml at 20% parasitemia in 3% BSA in RPMI containing uninfected red blood cells. The cells were then pelleted, and the supernatant was replaced with either soluble CSA as a control (100 μg/ml) or antibodies diluted in 1× PBS (DBPII affinity-purified IgG, 100 μg/ml; SD1ss affinity-purified IgG, 90 μg/ml; total IgG from unexposed Colombians, 90 or 100 μg/ml to match concentration of affinity-purified IgG). The samples were then incubated for 30 min at RT, and 20 μl was added to the CSA-coated spots. The plates were incubated for 15 min at RT. Plates were then placed on a rocker in a position such that PBS could be added to the lower portion of the plate and not come into contact with the spots. PBS (1×; 19 ml) was added to each plate, and the rocker speed was slowly increased, while a further 6 ml of 1× PBS was slowly added to the plate. The plates were washed for 8 min on the rocker before the PBS was aspirated from the plates, and the remaining cells were fixed by slowly adding 10 ml of 1.5% glutaraldehyde and incubating at RT for 10 min. The cells were stained with 10 ml of 5% Giemsa stain for 5 min and washed twice with 10 ml of deionized water. To quantify the number of parasites on each spot, the entire spot was imaged using an Evos FL Auto microscope (Invitrogen) with a 4×/0.13 phase lens objective. ImageJ was used to quantify the number of iRBCs bound to each spot. All experiments include replicates across multiple plates.

### Homology modeling.

The 3D7 DBL5ε homology model was created as described previously (75). Briefly, the multiple alignment was submitted to the HHpred server (76), and the best hit was selected based on score and structure resolution (VAR2CSA DBL3X, PDB ID 3BQK). The model was then validated by submission to the ProQ server (77), and PyMOL was used to generate figures (78).

### Statistical analysis.

Data were plotted using Prism software (version 8; GraphPad). Seroreactivity to different antigens was correlated using Spearman rank correlation (Fig. 3 and 4). Comparisons of parasite counts in IBAs were made using Student’s *t* test (Fig. 5C and D), and comparisons of competition ELISA data were made using one-way analysis of variance (ANOVA) with multiple-comparison tests (Fig. 6C to F and Fig. 7A).
