# Supplementary figures and images for: Antibodies to Cryptic Epitopes in Distant Homologues Underpin a Mechanism of Heterologous Immunity between Plasmodium vivax PvDBP and Plasmodium falciparum VAR2CSA
Source: mBio. 2019 Oct 8;10(5):e02343-19. doi: 10.1128/mBio.02343-19 (PMC6786876; doi:10.1128/mBio.02343-19)

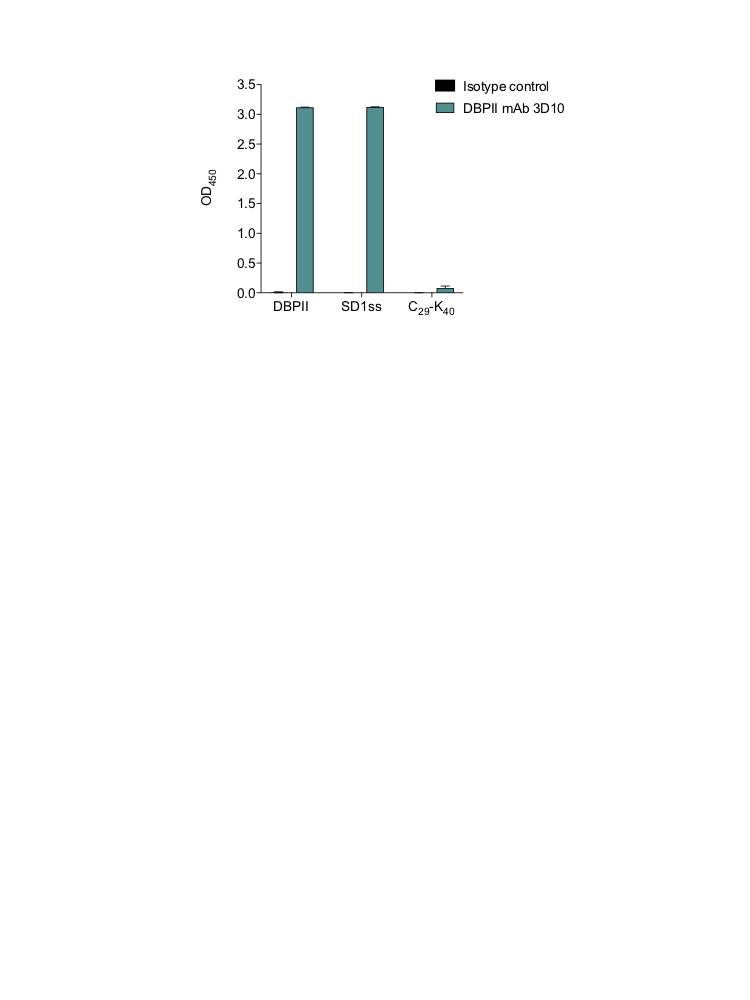

Supplement: FIG S1 [file mBio.02343-19-sf001.tif]

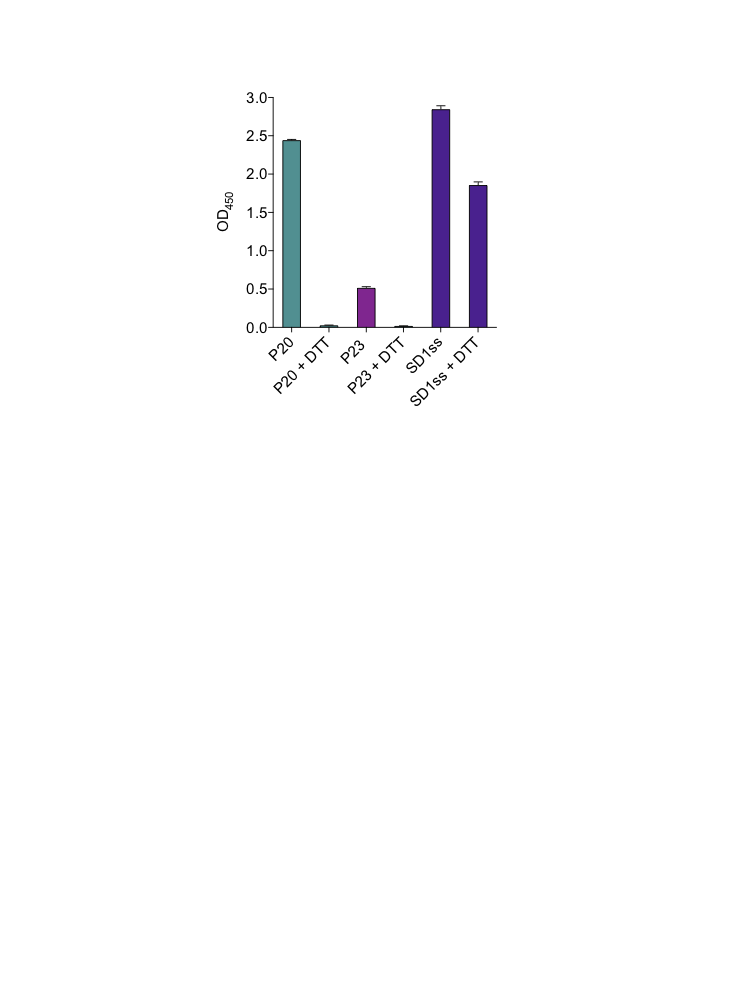

Supplement: FIG S2 [file mBio.02343-19-sf002.tif]
