# Supplementary material for: Antibodies to Cryptic Epitopes in Distant Homologues Underpin a Mechanism of Heterologous Immunity between Plasmodium vivax PvDBP and Plasmodium falciparum VAR2CSA
Source: mBio. 2019 Oct 8;10(5):e02343-19. doi: 10.1128/mBio.02343-19 (PMC6786876; doi:10.1128/mBio.02343-19)
Supplement: TABLE S1 [file mBio.02343-19-st001.pdf]

Supplementary Table 1. ELISA reagent details

Antigen	Antigen concentration (µg/mL)	Primary antibody	Primary antibody dilution	Secondary antibody dilution
**SD1ss**	1.0	3D10	Titration	1/3,000
		Sal 1 mouse IgG	5.0 µg/mL	
		Human sera	1/200	1/30,000
		Human IgG	5.0 µg/mL	
		Anti-VARCSA rabbit serum	1/750	1/3,000
**DBPII**	0.5	3D10	0.43 µg/mL	1/3,000
		Sal 1 mouse IgG	0.10 µg/mL	
		Human sera	1/100	1/40,000
		Human IgG	5.0 µg/mL	
**C_{29} – K_{40}**	5.0	3D10	4.3 µg/mL	1/3,000
**VAR2CSA**	1.0	3D10	8.6 µg/mL	1/3,000
		Sal 1 mouse IgG	5.0 µg/mL	
	0.5	Mouse serum	1/250	1/3,000
		Human sera	1/500*	1/6,000
		Human sera	1/1000	1/15,000
		Human IgG	5.0 µg/mL	
		Anti-VARCSA rabbit serum	1/750	1/3,000
		Anti-VARCSA rabbit serum	1/200**	1/3,000
**EBP2**	0.5	Human sera	1/200	1/40,000
		Human IgG	5.0 µg/mL	
**PfMSP1**	0.5	Human IgG	5.0 µg/mL	1/40,000
**DBL5ε**	0.5	3D10	4.0 µg/mL	1/3,000
**DBL5ε peptides**	5.0	3D10	0.86 µg/mL	1/3,000
		Human sera	1/200	1/40,000
		Anti-VARCSA rabbit serum	1/750	1/3,000

*Human sera were tested against VAR2CSA at a dilution of 1/500 in ELISAs performed in Brazil (Figure 2D).
**Rabbit sera were used at a dilution of 1/200 for antibody competition ELISAs (Figure 6C-E).
